# Crystal Polymorph Selection Mechanism of Hard Spheres Hidden in the Fluid

**DOI:** 10.1021/acsnano.3c02182

**Published:** 2023-04-21

**Authors:** Willem Gispen, Gabriele M. Coli, Robin van Damme, C. Patrick Royall, Marjolein Dijkstra

**Affiliations:** † Soft Condensed Matter &and Biophysics, Debye Institute for Nanomaterials Science, Utrecht University, Princetonplein 1, 3584 CC Utrecht, Netherlands; ‡ Gulliver UMR CNRS 7083, ESPCI Paris, Université PSL, 75005 Paris, France; ∥ H. H. Wills Physics Laboratory, University of Bristol, Tyndall Avenue, Bristol BS8 1TL, United Kingdom; § School of Chemistry, University of Bristol, Cantock’s Close, Bristol BS8 1TS, United Kingdom

**Keywords:** colloids, nucleation, polymorph selection, crystallization mechanism, hard spheres

## Abstract

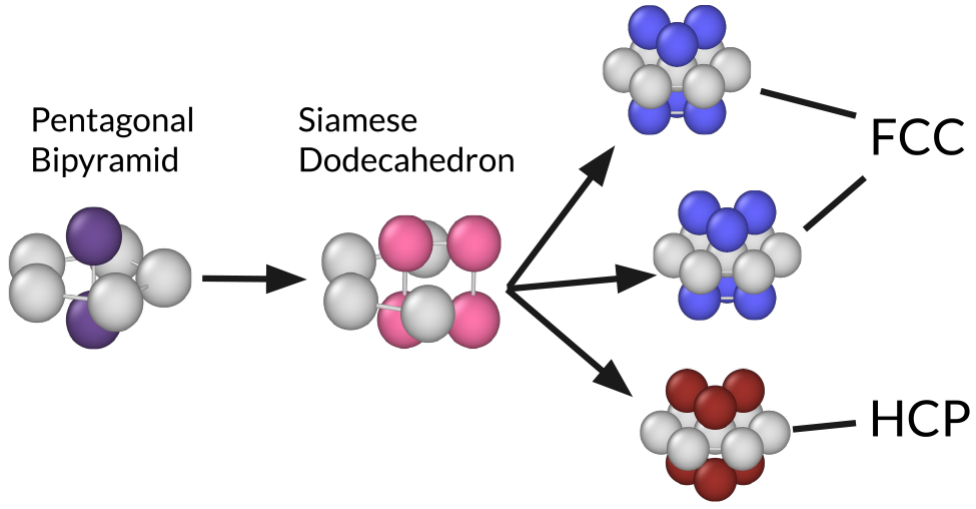

Nucleation plays a critical role in the birth of crystals and is associated with a vast array of phenomena, such as protein crystallization and ice formation in clouds. Despite numerous experimental and theoretical studies, many aspects of the nucleation process, such as the polymorph selection mechanism in the early stages, are far from being understood. Here, we show that the hitherto unexplained excess of particles in a face-centered-cubic (fcc)-like environment, as compared to those in a hexagonal-close-packed (hcp)-like environment, in a crystal nucleus of hard spheres can be explained by the higher order structure in the fluid phase. We show using both simulations and experiments that in the metastable fluid phase, pentagonal bipyramids, clusters with fivefold symmetry known to be inhibitors of crystal nucleation, transform into a different cluster, Siamese dodecahedra. These clusters are closely similar to an fcc subunit, which explains the higher propensity to grow fcc than hcp in hard spheres. We show that our crystallization and polymorph selection mechanism is generic for crystal nucleation from a dense, strongly correlated fluid phase.

## Introduction

Understanding nucleation is essential in various research fields including determining the molecular structure of proteins through crystallization, drug design in the pharmaceutical industry, and ice crystal formation in clouds. The latter is the largest unknown in the earth’s radiative balance and thus is crucial in the context of climate change and weather forecasts. Nucleation is also important in the crystallization of colloidal and nanoparticle suspensions, which have application perspectives in catalysis, optoelectronics, and plasmonics.^1,2^

However, studying nucleation in molecular systems is extremely challenging, as it is a stochastic and rare process. Additionally, the crystal nuclei sizes are often rather small, and the nuclei grow extremely quickly once they exceed their critical size. Moreover, in most substances, different crystal polymorphs may compete during nucleation, which is particularly important in pharmaceutical sciences and applications. Crystallization of the “undesired” polymorph may lead to neurodegenerative disorders such as Alzheimer’s disease or eye cataract or to reduced solubility/efficacy, and even toxicity of certain drug compounds.^3,4^

Recently, impressive strides have been made in the experimental observation of early stage crystal nucleation. Atomic-resolution in situ electron microscopy has shown the observation of different nucleation pathways leading to different crystal polymorphs of proteins,^5^ prenucleation clusters in metal organic frameworks,^6^ early stage nucleation pathways of FePt nanocrystals that go beyond classical nucleation theory and nonclassical scenarios,^7^ amorphous precursors in protein crystallization,^8^ featureless and semiordered clusters of NaCl nanocrystals,^9^ and reversible disorder–order transitions of gold crystals.^10^ These recent observations challenge current nucleation models and highlight the need for a better theoretical understanding of the crystallization pathways at the earliest stages of nucleation when particles start to order from the metastable fluid phase and select the emerging crystal polymorphs.

Colloidal suspensions are suitable experimental systems for investigating locally heterogeneous phenomena, such as early stage nucleation. The larger sizes and slower time scales of colloidal particles enable direct observation of the nucleation mechanisms.^11,12^ However, even for hard spheres, which are arguably the simplest colloidal model system, the polymorph selection mechanism has not yet been revealed. In a hard-sphere system, the hexagonal-close-packed (hcp) crystal is metastable with respect to the face-centered-cubic (fcc) crystal, but the free-energy difference between the two structures is tiny (approximately 10^–3^*k*_B_*T* per particle).^13,14^ Therefore, one might expect to find approximately 50% occurrence of fcc- and hcp-like particles in the crystal nucleus of hard spheres. However, this prediction is not realized in experiments^11,12,15−17^ nor in simulations,^18−22^ which both show a hitherto unexplained predominance of fcc particles in the final crystal phase.

In this article, we investigate the early stages of nucleation of hard spheres using simulations and particle-resolved experiments to shed light on the selection mechanism of the crystal polymorph. We study the structural transformations in the supersaturated fluid phase that finally lead to crystal nucleation. We find that the crystal embryo exhibits a preference toward fcc-like stacking due to its similarity with the local geometric motifs in the particle arrangements present in the fluid phase.

## Results and Discussion

### The Free-Energy Barrier for Nucleation

According to the Stranski–Totomanow conjecture, polymorph selection is governed by the lowest free-energy barrier for nucleation. Sanchez-Burgos et al.^23^ argued that fcc is preferred for hard spheres because it has a nucleation barrier marginally lower than that of hcp. They also found that a random-stacked nucleus has a higher nucleation barrier than fcc, even though random stacking commonly occurs in nucleation. This apparent discrepancy begs the crucial question whether the polymorph selection mechanism has a kinetic or thermodynamic origin. To answer this question, we calculate the Gibbs free energy βΔ*G*(*n*_fcc_, *n*_hcp_) for the formation of a crystal cluster consisting of *n*_fcc_ fcc-like particles and *n*_hcp_ hcp-like particles using the umbrella sampling technique; see the Methods section for the technical details.

**Figure 1:**
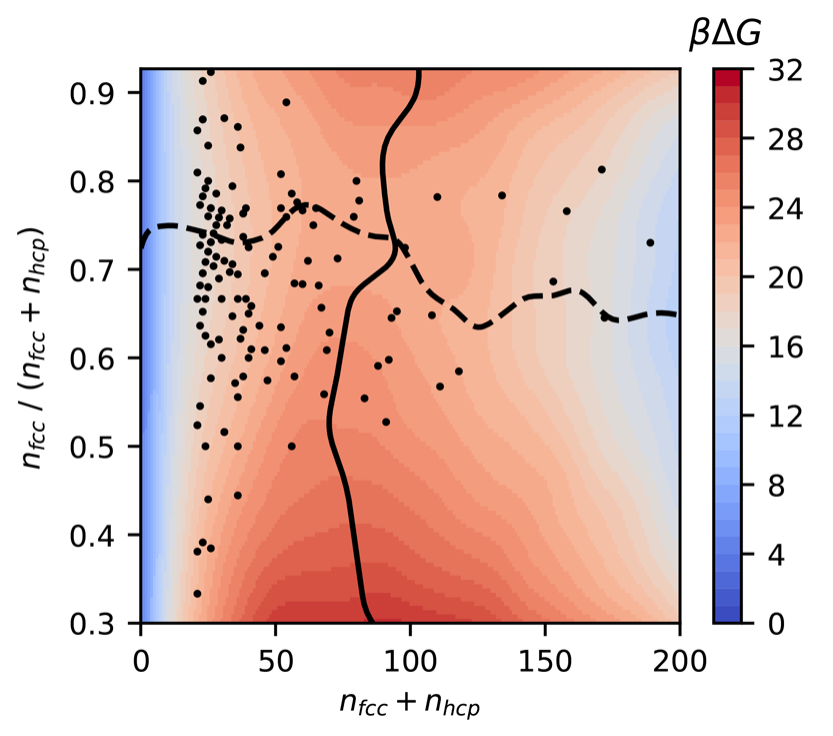
Thermodynamic propensity toward mixed fcc-hcp crystal nuclei in the early stages of crystal nucleation of hard spheres. Gibbs free-energy barrier as a function of the number of fcc and hcp particles *n*_fcc_ + *n*_hcp_ and composition *n*_fcc_/(*n*_fcc_ + *n*_hcp_). The dots are the composition of crystal nuclei found in our experiments. For both simulations and experiments, the composition of crystal nuclei is determined with the classification scheme described in the Methods Section. The dashed line represents the minimum free-energy path for nucleation, and the solid line represents the critical nucleus size as a function of composition.

In Figure 1, we plot βΔ*G* of a crystalline nucleus as a function of the size *n*_fcc_ + *n*_hcp_ and composition *n*_fcc_/(*n*_fcc_ + *n*_hcp_). The lowest free-energy path on this surface, represented by the dashed line, shows that the crystal nucleus has an excess of fcc-like particles in the early stages of nucleation and that the critical nucleus consists of about 70% fcc-like particles. In the SI, we show that this ratio is not strongly dependent on a specific polymorph detection method, as a variety of methods all find 60%–80% fcc-like particles.

We confirm the same phenomenon in an experimental system consisting of poly(methyl methacrylate) particles. We obtained particle coordinates by tracking them using confocal microscopy. More details on the experiments can be found in the Methods Section. We plot the size and composition of crystal nuclei found in our experiments, showing that the crystal nucleus exhibits a notable excess of fcc-like particles already in an early stage of nucleation.

To provide a rigorous comparison with classical nucleation theory, we also plot the critical nucleus size n* with a solid line. Intriguingly, we observe that the critical nucleus size n* remains nearly constant irrespective of the composition *n*_fcc_/(*n*_fcc_ + *n*_hcp_), while the nucleation barrier ΔG* = Δ*G*(n*) strongly depends on the composition. According to classical nucleation theory, n* and ΔG* should be directly proportional, as , where Δμ denotes the supersaturation of the crystal phase with respect to the fluid phase. As the difference in supersaturation between fcc and hcp is very small, the variation of the nucleation barrier with composition cannot be explained by this classical relation. This finding, combined with the observation that fcc is already preferred for very small nucleus sizes, strongly suggests that thermodynamic considerations based solely on surface tension and classical nucleation theory may not provide the full picture and that the metastable fluid deserves further scrutiny.

### Topological Structure of the Metastable Fluid

It has been suggested that polymorph selection is hidden in the fluid phase.^21^ To study this, we perform molecular dynamics (MD) simulations of a supersaturated fluid of hard spheres. We investigate the structure of the fluid by identifying the geometric motifs of particle assemblies locally present in the fluid phase by using the topological cluster classification (TCC) algorithm.^24^ Hereafter, we refer to these local particle assemblies as *clusters*, as their bond topologies are identical to certain minimum-energy clusters. We first identify rings of three, four, and five particles and then define basic clusters as rings with one or two additional particles, forming pyramids or bipyramids. In total, we distinguish 40 topological clusters composed of these basic clusters. To define a single-particle property, we determine the number of clusters of each type that each particle belongs to. We examine each particle configuration independently and counted the number of clusters. We use the TCC because it offers a more interpretable classification of local structure. Unlike order parameters based on rotational symmetry,^21^ which may be difficult to interpret, a topological cluster directly corresponds to a real space arrangement of particles. Furthermore, the TCC does not assume the presence of crystal-like symmetries, providing us with greater resolution on intermediate structures that may not be present in bulk crystal phases.

**Figure 2:**
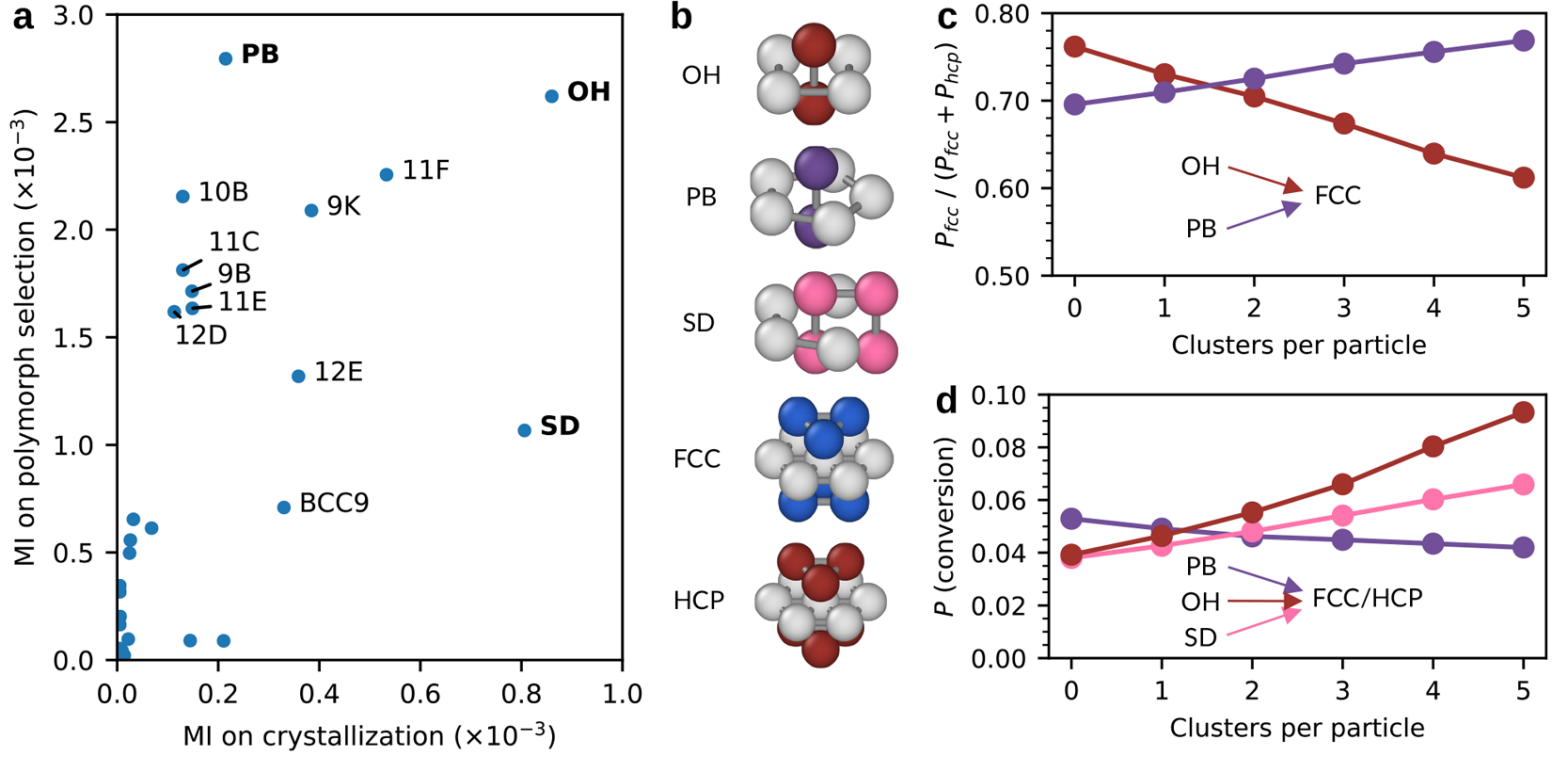
The effect of topological clusters on crystallization and polymorph selection in the metastable fluid. (a) Mutual information (MI) of topological clusters on crystallization and polymorph selection. Each label (e.g., 11F) refers to a different cluster as identified by the topological cluster classification algorithm.^24^ Clusters with low MI are not labeled. (b) Typical arrangements of particles in an OH, PB, SD, fcc, and hcp clusters. The ring particles in each cluster are colored light gray. (c) Relative probability to convert to fcc with respect to hcp as a function of the number of OH or PB clusters a particle is part of. (d) Probability to convert to fcc or hcp as a function of the number of PB, OH, or SD clusters it belongs to.

In Figure 2 we show the impact of these topological clusters on crystallization and polymorph selection in the metastable fluid. First, we study whether the clusters affect crystallization by calculating the probability that particles become crystalline, i.e., become part of either an fcc or hcp cluster. Second, we examine whether the clusters affect polymorph selection by determining the relative probability of particles becoming part of fcc or hcp. Note that the fcc and hcp clusters have a short lifetime in the metastable fluid, as they are smaller than the critical nucleus size. Therefore, we use a small time interval  to measure the conversions. To quantify the effect of each individual cluster on these probabilities, we computed the mutual information (MI) between the number of clusters a particle belongs to and the corresponding probabilities. In Figure 2a, we present a scatter plot of the MI on crystallization and polymorph selection. Three clusters, the octahedron (OH), the pentagonal bipyramid (PB), and the Siamese dodecahedron (SD), stand out.

In Figure 2b we present the geometries of these clusters. The OH is composed of a square ring with two additional particles forming the peaks of the pyramid. Similarly, the PB is a pentagonal ring with two additional particles. The SD is similar to PB, but one of the ring particles is replaced by two particles, one below and one above the ring. Finally, we depict the fcc and hcp clusters consisting of a central particle with its twelve nearest neighbors arranged as in bulk fcc or hcp.

As the OH and PB bipyramids exhibit the highest mutual information with polymorph selection, we show in Figure 2c the relative probability of converting to fcc with respect to hcp as a function of the number of these clusters a particle is part of. We observe that the relative probability to convert to fcc decreases as the number of OH increases. In bulk fcc and hcp, the number of OH is equal, suggesting that OH has an equal preference for fcc and hcp. Therefore, the decrease in the relative probability with the number of OH indicates a drift toward 50%. On the other hand, the relative probability to convert to fcc increases as the number of PB clusters a particle belongs to increases. This finding is peculiar as the fivefold symmetry of PB is incommensurate with the sixfold symmetry of the fcc crystal.

Similarly, we assessed the impact of OH, PB, and SD on crystallization. In Figure 2d, we show the probability of particles to convert to either fcc or hcp as a function of the number of clusters they belong to. We observe that the probability to crystallize decreases with the number of PB clusters a particle belongs to, which is consistent with previous findings that fivefold symmetry suppresses crystallization.^21,25−27^ In fact, our observation that PB clusters prefer to convert to fcc suggests that this inhibiting effect of fivefold symmetry on crystallization is due to the reduced conversion to hcp. In contrast, the probability of crystallization increases with the number of SD and OH clusters a particle is part of, with these clusters showing the strongest promotion of crystallization. For instance, the conversion probability of a particle that is part of five OH clusters has a conversion probability of more than twice that of a particle not belonging to any OH cluster.

### Topological Structure of Crystal Nuclei

**Figure 3:**
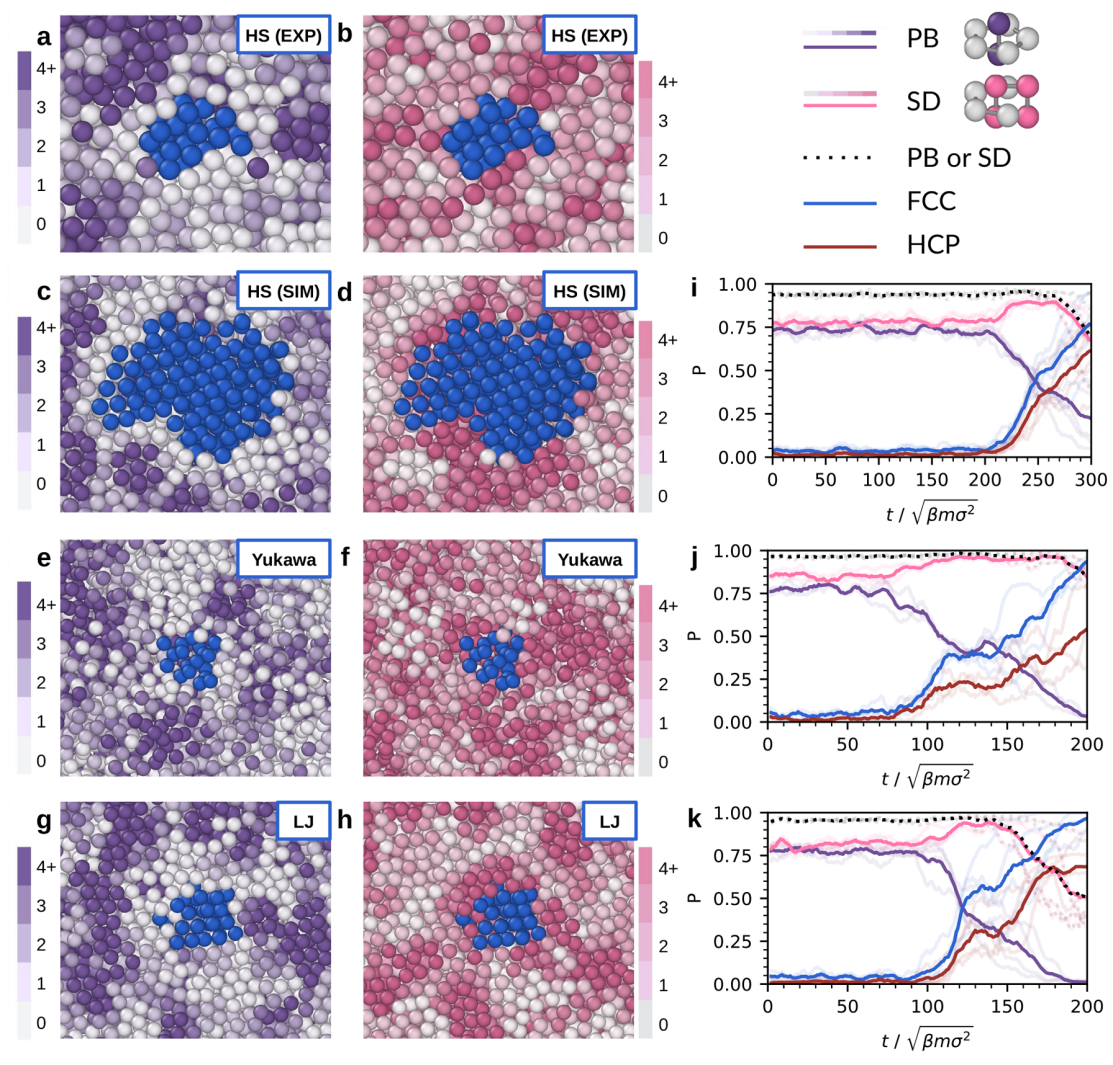
The role of PB and SD clusters during crystal nucleation from strongly correlated, dense fluids. (a,b) PMMA spheres imaged with confocal microscopy. (c,d,i) Nearly hard spheres interacting with a WCA potential. (e,f,j) Charged spheres interacting with a Yukawa potential. (g,h,k) Spheres interacting with a Lennard-Jones potential. (a–h) On the left, we show cut-through images of crystal nuclei. The core of the crystal nucleus is colored blue, while the rest of the particles is colored following the scale bar on the left (a,c,e,g) or right (b,d,f,h) depending on the number of PB (a,c,e,g) or SD (b,d,f,h) clusters each particle belongs to. (i–k) On the right, we show the fraction of particles belonging to different clusters as a function of time (solid lines), averaged over multiple different simulated spontaneous nucleation events (transparent lines). The fractions are averaged over 5 (i), 3 (j), and 3 (k) different nucleation events, respectively. Note that a particle can be part of multiple clusters at the same time, and therefore the corresponding fractions add up to a value which is higher than one.

These results suggest that the predominance of fcc is a result of the fivefold structure of the fluid. But how do these clusters actually behave *during* nucleation? To investigate this, we calculated the number of PB, SD, fcc, and hcp clusters during nucleation. In Figure 3a,b, we present cut-through images of a small crystal nucleus in an experimental sample. We color a particle blue if it belongs to at least three fcc and hcp clusters. This enables us to clearly identify the structure of the surface of the nucleus. We color the particles on the surface and in the surrounding fluid with varying shades of purple and pink depending on the number of SD or PB clusters they belong to. Despite the high density of SD and PB clusters present throughout the fluid, Figure 3a,b shows that the density of these clusters is spatially heterogeneous. Specifically, we observe that the crystal nucleus is surrounded by a high density of SD clusters, whereas the opposite trend is found for the PB clusters, as the PB clusters are depleted near the surface of the crystal nucleus.

To demonstrate the universality of this phenomenon, we examined in simulations the crystal nucleation of three different systems: hard spheres, charged spheres, and Lennard-Jones particles. In the charged-sphere system, we used a screened-Coulomb (Yukawa) potential that has a longer and softer repulsive interaction compared with the hard-sphere interaction. The Lennard-Jones system has both attractive and repulsive interactions. We visualize the crystal nuclei in Figure 3c–h, using the same color coding as for the experimental sample in Figure 3a,b. All these systems exhibit a similar heterogeneous structure consisting of high and low density regions of SD and PB clusters in the fluid phase and a crystal nucleus that is surrounded by a high density of SD clusters and a low density of PB clusters. For a quantitative comparison between experiments and simulations, see the SI. Furthermore, our analysis in the SI demonstrates that a high density of SD clusters and a low density of PB clusters are also present near *planar* solid–fluid interfaces. These findings suggest that the observed behavior is a universal phenomenon that arises from geometric constraints on particle arrangements and is not specific to a particular type of interaction potential.

To understand the role of the topological clusters in the crystallization mechanism, we plot in Figure 3 the fraction of particles belonging to SD (pink line), PB (purple), fcc (blue), and hcp (brown) clusters as a function of time, averaged over multiple spontaneous nucleation trajectories obtained in simulations. Notably, we observe the same behavior in all of these systems. Figure 3i–k reveals that while the fraction of crystalline particles is initially low in the metastable fluid phase, the fraction of fcc clusters is clearly higher than that of hcp clusters at this stage in the fluid phase. The fluid phase already contains 70% fcc and 30% hcp, which agrees well with our umbrella sampling results shown in Figure 1. Furthermore, we observe that the populations of particles in both the SD and PB clusters are already high before crystallization sets in, indicating that the metastable fluid exhibits strong spatial correlations due to packing constraints. At the onset of crystallization, the fraction of PB clusters decreases, reminiscent of the decrease in fivefold symmetry observed at the onset of crystallization.^27^ More surprisingly, our analysis reveals that the number of SD clusters increases during the early stages of crystallization, which later decreases to a lower value at the end of the crystallization process, as the fcc structure does not contain SD clusters. Notably, the SD cluster is the only cluster that shows nonmonotonic behavior during nucleation (see Figure S4 of the SI).

To further examine the relationship between SD and PB clusters, we measure the combined fraction of particles belonging to either SD or PB clusters as a function of time (dotted line in Figure 3i–k). Our results indicate that the combined fraction remains constant not only in the metastable fluid phase but also during the early stages of crystallization. We also note that the combined fraction fluctuates less than the individual fractions of the SD and PB clusters. These observations suggest that the increase in SD clusters is a direct consequence of a decrease in PB clusters. Moreover, the constant combined fraction and much smaller fluctuations indicate a reversible conversion between the PB and SD clusters.

In the SI, we quantify the conversions among PB, SD, fcc, and hcp clusters, providing further evidence that the SD cluster acts as an intermediate state between PB clusters and fcc/hcp clusters. Additionally, we show that the findings presented in Figure 3 remain unchanged when we change the dynamics of hard spheres to hydrodynamics or event-driven dynamics. These results lend strong support that the observed crystallization and polymorph selection mechanism is generic for crystal nucleation from a highly correlated, dense fluid phase.

### The Nucleation Mechanism

**Figure 4:**
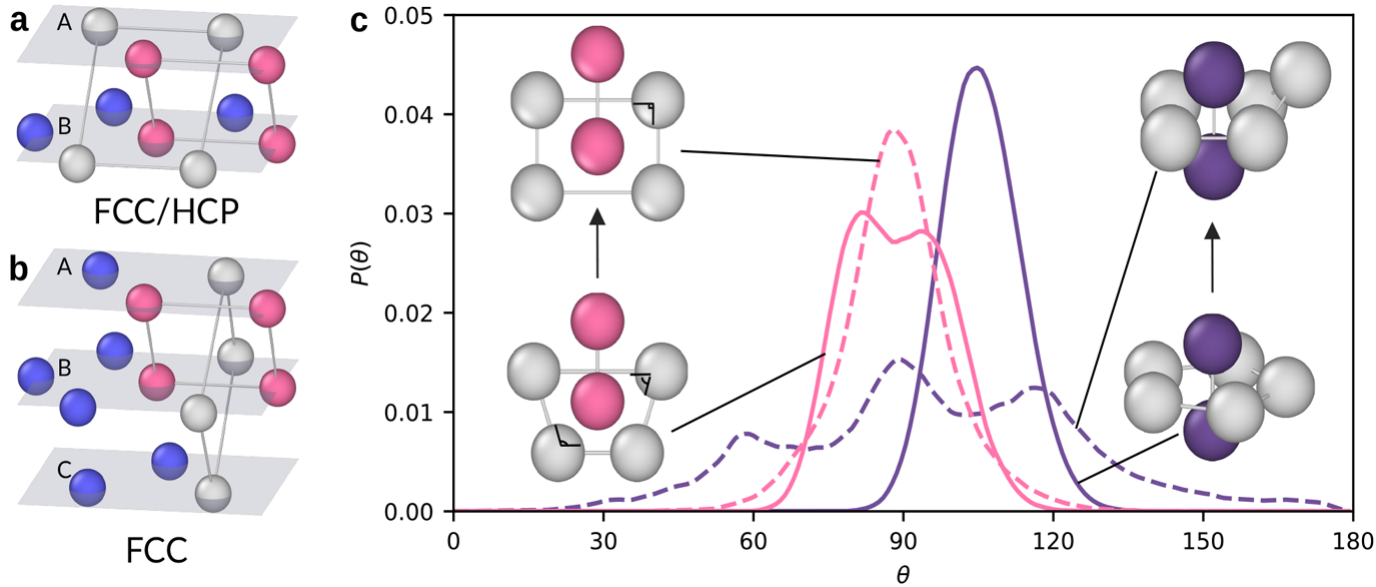
Transition of SD and PB clusters to fcc subunits. (a) Geometry of the SD cluster (gray and pink particles) parallel to two hexagonal layers of an fcc and hcp crystal. (b) Geometry of the SD cluster perpendicular to the hexagonal layers, which occurs only in fcc. (c) Probability distributions of the internal angles θ of the ring particles of SD and PB clusters before and after the transition to an fcc subunit. The typical arrangements of particles in SD (left) and PB (right) clusters before and after the transition are also shown, with the ring particles colored in gray.

To elucidate how the fivefold structure of the PB and SD clusters becomes integrated into the fcc or hcp crystal lattice, we note that the four particles comprising the pentagonal ring of an SD cluster form a trapezoidal arrangement with two acute and two obtuse angles, as shown in the bottom left of Figure 4c. When these particles arrange in a square configuration, the SD cluster can be identified as a subunit of an fcc or hcp crystal, as illustrated in Figure 4a,b, where the particles are denoted with the same colors to facilitate comparison. These “squared” SD clusters can fully tile both the fcc and hcp lattices without needing any additional particles. However, the “squared” SD cluster fits into the fcc crystal in two ways, parallel (Figure 4a) and perpendicular (Figure 4b) to the hexagonal layers, whereas it fits only in one way in the hcp crystal, namely parallel to the hexagonal layers (Figure 4a). Thus, the SD cluster has greater orientational freedom in forming the fcc lattice, resulting in a 2-to-1 preference for fcc over hcp, which is close to the 70% ratio that we observe in our simulations. We note that this 2-to-1 preference results from the greater symmetry of the fcc crystal, which has been suggested to favor fcc during nucleation.^28^

Given the topological similarity between the SD cluster and the fcc lattice, we speculate that the attachment of fluidlike particles to the solid nucleus proceeds via SD clusters, where the four particles in the pentagonal ring transform from a trapezoidal to a square arrangement. In this way, the SD clusters are fully incorporated into the fcc lattice. To test this hypothesis, we identify the SD clusters that become part of an fcc or hcp cluster during nucleation and measure the distribution of the four angles of the trapezoidal arrangement of the four particles in the pentagonal ring of the SD clusters. As shown in Figure 4c, the distribution is bimodal with peaks at angles smaller and larger than 90°, representing the trapezoidal arrangement, before the transition. However, after the transition, the distribution becomes unimodal with a single peak at around 90°, indicating a square pattern. This transition is further illustrated in Figure 4c, which shows two representative SD clusters before and after the transformation.

To further validate our proposed nucleation mechanism, we measured the distribution of the internal angles of the five particles in the ring of the PB cluster. Before the transition, the distribution is centered around the expected 108° for a fivefold ring. After the transition, we observe a split into three peaks, with the peaks centered around 60°, 90°, and 120°, providing compelling evidence for the transformation of the PB cluster as illustrated in Figure 4c.

This result strongly reinforces our key finding that the fivefold PB clusters, which are abundant in the fluid phase and known to inhibit crystal nucleation, transform into SD clusters and that the SD cluster-mediated attachment of particles to the growing nucleus proceeds via a simple rearrangement of particles into fcc subunits. Hence, the propensity to grow fcc is higher than that for hcp, indicating that the polymorph selection mechanism in hard spheres is already hidden in the higher order structure of the fluid phase.

## Conclusions

In conclusion, we unveil the underlying crystallization and polymorph selection mechanism in a highly correlated, dense fluid phase such as hard spheres during the early stages of nucleation both in simulations and experiments. We demonstrate that the supersaturated fluid is highly dynamic, with reversible transformations between fivefold pentagonal bipyramid and Siamese dodecahedron clusters. Most notably, the Siamese dodecahedra exhibit a close similarity with an fcc subunit, providing an explanation for the as-of-yet-unexplained higher propensity of fcc over hcp in hard spheres. Moreover, we reveal that the polymorph selection mechanism has not only a geometric origin, hidden in the higher-order correlations of the fluid phase, but also a thermodynamic origin, with the lowest free-energy pathway favoring a higher number of fcc-like particles in the early stages of nucleation. Our findings offer valuable insights for controlling nucleation pathways and crystal polymorphs in highly correlated fluid phases and provide a framework for studying nucleation in other systems.

## Methods

### Umbrella Sampling Simulations

To investigate the thermodynamic propensity toward fcc-like or hcp-like ordering during the nucleation process, we use umbrella sampling (US)^29^ to calculate the nucleation barrier of a system of hard spheres at a pressure of *βPσ*^3^ = 17.0. This pressure corresponds to a packing fraction of 0.5352 for the fluid phase.^20^ We note that under these conditions, the absolute nucleation rate as predicted by US is in reasonable agreement with experiments.^20^ Similar to previous literature,^20,30^ we identify the nucleus by using the dot product *d*_6_ of local spherical harmonics expansions with *l* = 6 to define solid-like bonds as those bonds between particle pairs (*i*, *j*) for which *d*_6_(*i*, *j*) > 0.7 and define solid-like particles as those that have at least 7 of such solid-like bonds. Particle neighbors are defined by using a distance cutoff of *r*_*c*_ = 1.4σ. The nucleus is then the largest set of solid-like particles that are connected by solid-like bonds. To disentangle fcc-like and hcp-like order, we subsequently classify solid-like particles as fcc-like and hcp-like based on their value of the Steinhardt bond order parameter *w*_4_: particles with *w*_4_ < 0 are fcc-like and those with *w*_4_ ≥ 0 are hcp-like.^31^ The number of such particles are *n*_fcc_ and *n*_hcp_, respectively, and we use these to define the US biasing potential:
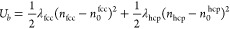
where both coupling constants *λ*_fcc_ and *λ*_hcp_ are set to an equal value of *βλ*_fcc_ = *βλ*_hcp_ = 0.05. This allows us to sample the two-dimensional Gibbs free-energy difference βΔ*G*(*n*_fcc_, *n*_hcp_) that is the nucleation barrier as a function of the number of fcc-like and hcp-like ordered particles.

We initialize each US window (*n*_0_^fcc^, *n*_0_^hcp^) from a configuration with a nucleus with approximately *n*_fcc_ ≈ *n*_0_^fcc^ and *n*_hcp_ ≈ *n*_0_^hcp^. For very small nuclei up to *n* = *n*_fcc_ + *n*_hcp_ ∼ 20, we measure the full cluster size distribution instead of only the size of the largest cluster, as the probability of multiple small nuclei appearing simultaneously can be significant. We implement the US scheme by adding additional Monte Carlo bias moves that accept or reject trajectories based on the US bias potential on top of a hard-particle Monte Carlo (HPMC) simulation implemented using HOOMD-blue’s HPMC module.^32,33^ Bias moves are performed every MC cycle in order to also sample regions of the free-energy landscape where the gradient is large. Finally, we reconstruct the nucleation barrier by using the weighted histogram analysis method (WHAM),^34^ specifically by using the algorithm provided by ref (35).

The minimum free-energy path for nucleation, plotted in Figure 1, is calculated by weighting the composition of nuclei of a given size with the modified Boltzmann factor exp(− 5βΔ*G*(*n*_fcc_, *n*_hcp_)). The critical nucleus size as a function of its composition, also plotted in Figure 1, is calculated by weighting the size of all nuclei with a certain composition with the inverse modified Boltzmann factor exp(5βΔ*G*(*n*_fcc_, *n*_hcp_)). We find that these smooth versions of the minimum and maximum functions are more numerically stable against the statistical errors in our US simulations.

### Topological Cluster Classification

We require an algorithm that is capable of interpretably quantifying the structure in the metastable fluid. To this end, we use the topological cluster classification (TCC) algorithm.^24^ The bonds between particles are detected by using a modified Voronoi construction method. The free parameter *f*_*c*_, controlling the amount of asymmetry that a four-membered ring can show before being identified as two three-membered rings, is set to 0.82.

### MD Simulations

In order to generate trajectories in which we observe spontaneous nucleation, we conducted MD simulations in the isothermal–isobaric (NPT) ensemble. For all systems, the temperature *T* and pressure *P* are kept constant via the Martyna–Tobias–Klein (MTK) integrator.^36^ The simulation box is cubic, and periodic boundary conditions are applied in all directions.

To simulate nearly hard spheres, we use a constant number *N* = 13500 of particles interacting via a Weeks–Chandler–Andersen (WCA) pair potential, which can straightforwardly be employed in Molecular Dynamics (MD) simulations and which reduces to the hard-sphere potential in the limit that the temperature *T* → 0. The WCA pair interaction *u*(*r*_*ij*_) is simply a Lennard-Jones potential cut-and-shifted at the minimum of its potential well and reads^37^
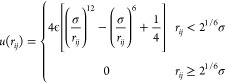
with *r*_*ij*_ = | **r**_*i*_ – **r**_*j*_| the center-of-mass distance between particle *i* and *j*, **r**_*i*_ the position of particle *i*, ϵ the interaction strength, and σ the diameter of each sphere. The steepness of the repulsion between the particles can be tuned by the temperature *k*_B_*T*/ϵ. We set *k*_B_*T*/ϵ = 0.025, which has been used extensively in previous simulation studies to mimic hard spheres.^38−41^ The reduced pressure is chosen as *βPσ*_eff_^3^ = 17.69, with the thermostat and barostat coupling constants *τ*_*T*_ = 1.0 *τ*_MD_ and *τ*_*P*_ = 1.0 *τ*_MD_, respectively, and  is the MD time unit. The time step is set to Δ*t* = 0.0001*τ*_MD_, which is small enough to ensure the stability of the simulations. We used the mapping described in refs,^39−41^ which results in each particle having an effective diameter σ_eff_ ≃ 1.097. With this mapping, the fluid phase has an effective packing fraction of 0.539. The MD simulations for nearly hard spheres are performed using the HOOMD-blue (highly optimized object-oriented many-particle dynamics) software.^32^

To simulate charged spheres, we use a pseudohard-core Yukawa potential. The total interaction is the sum of the pseudohard-core potential^42^ and a screened Coulomb (Yukawa) pair potential *u*_*Y*_(*r*_*ij*_):
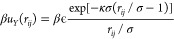
with contact value βϵ and screening length 1/*κσ*. For this system, we simulate *N* = 10^4^ particles with contact value βϵ = 81, screening length 1/*κσ* = 0.125, and pressure *βPσ*^3^ = 11.0. Under these conditions, the packing fraction of the fluid is η = *πσ*^3^*N*/6*V* = 0.203. We used a time step Δ*t* = 0.0025*τ*_MD_, with the thermostat and barostat coupling constants *τ*_*T*_ = 100.0Δ*t* and *τ*_*P*_ = 500.0Δ*t*, respectively. The MD simulations for charged spheres are performed using the LAMMPS molecular dynamics code.^43^

For the Lennard-Jones system, we simulate *N* = 10^4^ particles at a temperature *k*_B_*T*/ϵ = 1.0 and a pressure *Pσ*^3^/ϵ = 11.5. At this temperature and pressure, the liquid has a number density of *ρσ*^3^ = 1.04. The interaction potential was truncated and shifted at 2.5σ. We used a time step Δ*t* = 0.005*τ*_MD_, with the thermostat and barostat coupling constants *τ*_*T*_ = 100.0Δ*t* and *τ*_*P*_ = 500.0Δ*t*, respectively. The MD simulations for the Lennard-Jones system are also performed using the LAMMPS molecular dynamics code.

To average multiple nucleation trajectories, such as in Figure 3, we shift the trajectories in time such that the number of solid-like particles reaches 50 at the same time. Additionally, to focus on nucleation, we “zoom in” to a small cubic box containing 200 particles around the center of mass of the nucleus of 50 solid-like particles. Using this procedure, the different nucleation trajectories show qualitatively the same behavior. By plotting the different nucleation trajectories with transparent lines, we aim to give a rough indication of the variance in the data.

The images of topological clusters and crystal nuclei were produced by using the OVITO visualization software.^44^

### Experiments

We used poly(methyl methacrylate) (PMMA) particles of diameter 2.00 μm with a polydispersity of 4.0% as determined by static light scattering, which were fluorescently labeled with Rhodamine dye. The particles were dispersed in a density matching mixture of cis decalin and cyclohexyl bormide. Tetrabutyl ammonium bromide salt was used to screen the electrostatic charges. The resulting dispersions were imaged by using a Leica SP5 confocal microscope to obtain real space particle coordinates. Due to the residual electrostatic interactions, the effective hard sphere diameter is 1.02 times that of the physical diameter and thus, we quote experimental values in effective packing fractions. Further details are available in ref (45).

## Data Availability

The code used to generate and analyze the results of this paper is freely available at https://github.com/MarjoleinDijkstraGroupUU/Crystal-Polymorph-Selection-Mechanism-of-Hard-Spheres-Hidden-in-the-Fluid.

## Supporting Information Available

Gibbs free energy for nucleation in the *n*_fcc_–*n*_hcp_ plane; robustness with respect to polymorph detection method; robustness with respect to dynamics; conversions between PB, SD, fcc, and hcp clusters; behavior of all clusters during nucleation and near planar solid–fluid interfaces; quantitative comparison between experiments and simulations (PDF).
