# Supplementary material for: Crystal Polymorph Selection Mechanism of Hard Spheres Hidden in the Fluid
Source: ACS Nano. 2023 Apr 21;17(9):8807–14. doi: 10.1021/acsnano.3c02182 (PMC10173683; doi:10.1021/acsnano.3c02182)
Supplement: Supplementary file 1 — nn3c02182_si_001.pdf [file nn3c02182_si_001.pdf]

Supporting Information: Crystal polymorph selection mechanism of hard spheres hidden in the fluid

Willem Gispen[1],† Gabriele M. Coli[1],† Robin van Damme,† C. Patrick Royall,‡,¶,§ and Marjolein Dijkstra*,†

†*Soft Condensed Matter & Biophysics, Debye Institute for Nanomaterials Science, Utrecht University, Princetonplein 1, 3584 CC Utrecht, Netherlands*

‡*Gulliver UMR CNRS 7083, ESPCI Paris, Université PSL, 75005 Paris, France*

¶*H.H. Wills Physics Laboratory, Tyndall Avenue, Bristol, BS8 1TL, UK*

§*School of Chemistry, University of Bristol, Cantock's Close, Bristol, BS8 1TS, UK*

E-mail: m.dijkstra@uu.nl

The free-energy barrier for nucleation and polymorph detection

Gibbs free energy for nucleation in the $n_{\mathbf{fcc}} - n_{\mathbf{hcp}}$ plane

In Fig. 1 of the main text, we show the Gibbs free energy $\beta\Delta G$ for the formation of a crystal cluster as a function of the cluster size $n_{\mathrm{fcc}} + n_{\mathrm{hcp}}$ and cluster composition $n_{\mathrm{fcc}}/(n_{\mathrm{fcc}} + n_{\mathrm{hcp}})$. In Fig. S1a we show the nucleation barrier in the $n_{\mathrm{fcc}} - n_{\mathrm{hcp}}$ plane. Compared to Fig. 1, Fig. S1a displays a slightly larger range of nucleus sizes and compositions. Notably, for low

n_{fcc} and high n_{hcp}, the Gibbs free energy $\beta\Delta G$ becomes almost $40kT$, indicating that the nucleation of almost purely hcp-like crystal nuclei is very strongly suppressed.

In principle, the Gibbs free energy landscape $\beta\Delta G(n_{\mathrm{fcc}}, n_{\mathrm{hcp}})$ could depend on the polymorph detection method. This is because different polymorph detection algorithms generally give different results, so that the composition $(n_{\mathrm{fcc}}, n_{\mathrm{hcp}})$ of a crystal nucleus will also depend on the algorithm. To make sure our results are robust, we validate our results using different methods.

Polymorph detection with different bond order parameters

When using bond order parameters there are several choices to make, which affect the classification of the particles. These choices are related to the identification of the local neighborhood of a particle, the distinction of fluid-like and solid-like particles, and the subsequent distinction of different crystal polymorphs. In this section, we select different criteria for all these sub-tasks, and check the robustness of the classification outcome. Specifically, we check that all methods record a predominance of fcc-like ordering with respect to hcp-like ordering in the growing nucleus.

To this end, we start by using three different techniques to find the local neighborhood of a particle. The first two are based on a simple cutoff radius equal to 1.4σ and 1.5σ with σ the diameter of the particles, while the third is based on the solid-angle nearest-neighbor (SANN) algorithm.[1] Furthermore, in order to classify particles as solid-like or fluid-like, we use two different criteria. The first is implemented via a criterion on the averaged bond order parameter[2] $\bar{q}_6 > 0.31$, while the second is based on dot-products of the q_{6m}, as implemented in the Umbrella Sampling calculations (see Methods). Finally, to distinguish the different crystal polymorphs, we again use different strategies. In the first method, we employ the w_4 value, as in our Umbrella Sampling calculations (see Methods). Alternatively, we can also use the averaged bond order parameter $\bar{w}_4$.

Using all possible combinations to detect the local environment, distinguish between solid

and fluid-like particles, and classify different polymorphs, we obtain a total of 12 classification algorithms. We use all of these to analyze a single spontaneous nucleation trajectory obtained from simulations and compute the fraction of fcc-like and hcp-like particles. In Fig. S1b, we show the ratio of fcc-like particles computed via each classification scheme. We note that the first part of the nucleation event is noisy as the denominator, i.e. the number of particles belonging to the main cluster, is very small.

We clearly observe that our results are robust with respect to the choice of classification scheme, thereby providing confidence that the crystal nuclei are predominately composed of 60%-80% fcc-like particles.

Polymorph detection with the topological cluster classification

In Fig. 3e of the main text, we show that more particles are part of an FCC cluster than of an HCP cluster during nucleation. In the fluid, this ratio is approximately 70%. During nucleation, however, this ratio drops significantly, which is seemingly inconsistent with the Umbrella Sampling simulations.

The explanation is as follows. Most local structure detection algorithms, including our bond order parameter (BOP) method, classify a single particle on the basis of its neighboring particles. In contrast, the TCC classifies a collection of particles on the basis of their interconnecting bonds. For example, the FCC cluster comprises thirteen particles, and in Fig. 3e, all these thirteen particles are counted as being part of the FCC cluster. A method such as the common neighbor analysis,[3] polyhedral template matching,[4] or our BOP method, would only classify the *central* particle of this cluster as fcc-like. Since each FCC and HCP cluster has only one central particle, the number of clusters is equal to the number of central particles. Therefore, to compare the TCC with our BOP method, we need to count the number of FCC and HCP clusters, instead of how many particles are part of these clusters.

In Fig. S1c, we show the fraction of FCC clusters for the five different spontaneous nucleation trajectories analyzed in the main text. We observe that the fraction of FCC

clusters is still in the 60%-80% range. Therefore, polymorph detection with the TCC is consistent with our BOP method.

Influence of dynamics on the nucleation mechanism

In the main text, we focus on molecular dynamics simulations of nearly hard spheres. Here, we show that the behavior of SD and PB clusters during nucleation is reproducible under different dynamics, further supporting the robustness of our findings.

In Fig. S2a-c we show the nucleation of nearly hard spheres with hydrodynamics. The hard spheres are modeled with a Weeks-Chandler-Andersen (WCA) potential at an effective packing fraction $\eta_{\text{eff}} = \pi\sigma_{\text{eff}}^3 N/6V = 0.543$. The hydrodynamics is modeled using the stochastic rotation dynamics method. The raw nucleation data were obtained from Ref. [5] and kindly provided by Giulia Fiorucci.

In Fig. S2d-f we show the nucleation of plain hard spheres as obtained from event-driven molecular dynamics simulations. The packing fraction is $\eta = 0.54$. The raw nucleation data were obtained from Ref. [6] and kindly provided by Michiel Hermes.

For both systems, we reproduce the findings presented in Fig. 3 of the main text, for example: the crystal nucleus is surrounded by a higher density of Siamese Dodecahedra (SD) and a lower density of Pentagonal Bipyramids (PB); the number of SD clusters increases at the onset of nucleation, and decreases at a later stage in the crystallization process; the number of PB clusters decreases in the initial stage of nucleation; the fluid already has a high number of PB and SD clusters; and more particles are part of an FCC cluster than of an HCP cluster in the fluid phase and during nucleation. Therefore, these conclusions do not depend on the specific dynamics.

Siamese Dodecahedra as a bridge between the fluid and the solid

In the main text, we show that PB and SD clusters play an important role in the crystallization and polymorph selection mechanism. In this section, we further investigate the relation between SD, PB, FCC and HCP clusters.

Conversions between topological clusters

We first investigate the relation between SD and PB clusters. In Fig. 3, we observe that the fraction of particles that are part of either an SD or PB cluster fluctuates less than the individual fractions, suggesting that there is a reversible conversion between the two clusters. To prove that this is indeed the case, we measure the conversions between PB and SD clusters in the fluid and during nucleation. For this purpose, we define a PB cluster at time t to be converted to an SD cluster at time $t + \Delta t$ if (1) at time t, the PB cluster is not a subset of any SD cluster, and (2) at time $t + \Delta t$, the particles forming the PB cluster are all part of the same SD cluster. In the same vein, we define an SD cluster at time t to be converted to a PB cluster at time $t + \Delta t$ if (1) at time t, the SD cluster is not a superset of any PB cluster, and (2) at time $t + \Delta t$, the particles forming the SD cluster are a superset of a PB cluster. In Fig. S3a, we show the conversion probabilities, i.e. the number of conversions divided by the number of clusters. To measure the conversions, we use $\Delta t/\sqrt{\beta m \sigma^2} = 1.6$. From Fig. S3a, we see that there are indeed reversible conversions between PB and SD clusters. Conversions are already happening in the fluid, but during nucleation, the conversion probability from PB to SD increases, while the conversion probability from SD to PB decreases. This is in line with the observation that the overall number of SD clusters first increases and the number of PB clusters decreases.

In Fig. S3b, we plot similar conversion probabilities from SD to FCC and HCP. In this

case, we define an SD cluster at time t to be converted to an FCC cluster at time $t + \Delta t$ if (1) at time t, there is no FCC cluster that shares more than six particles with the SD cluster and (2) at time $t + \Delta t$, at least seven particles forming the SD cluster are now part of the same FCC cluster. We use the same definition for the SD to HCP conversion. We choose the threshold of six/seven shared particles because an SD cluster cannot be a complete subset of an FCC or HCP cluster. This can also be seen from Fig. 4: because the FCC and HCP clusters contain only the nearest neighbors of a central particle, they are too small to completely contain the slightly elongated SD cluster. With this definition, we indeed see that there are SD to FCC and SD to HCP conversions and that the conversion probability increases at the start of nucleation. Both in the fluid and during nucleation, the conversion probability from SD to FCC is higher than from SD to HCP. In the fluid, the relative probability to convert to FCC is again 70% on average. This is again in line with the observation that there are more FCC clusters in the fluid and supports the idea that SD clusters are involved in the polymorph selection mechanism. Furthermore, the observation that SD *convert* to FCC and HCP and are not already part of FCC and HCP, shows that the SD clusters are indeed an intermediate structure in the crystallization process.

Overlap between SD and FCC/HCP clusters

To further investigate the relationship between SD and FCC/HCP clusters, we measure the number of particles that these clusters share. To be precise, for each SD cluster, we measure how many of the constituent particles are also part of an FCC or HCP cluster. In Fig. S3c, we plot the fraction of SD clusters having no (0 shared particles), intermediate (between 1 and 6 shared particles), or large (7 or 8 shared particles) overlap with FCC or HCP clusters. In the fluid, most SD clusters have no overlap with FCC or HCP, which is expected, since FCC and HCP clusters are rare in the fluid. At the start of nucleation, the fraction of SD clusters with intermediate overlap with FCC or HCP increases, and this briefly becomes the dominant contribution. As the nucleus grows, more and more SD clusters

become incorporated into an FCC or HCP crystal lattice. Interestingly, the fraction of SD clusters with intermediate overlap is especially important during nucleation, which shows once again that the SD clusters are an intermediate structure on the surface of the crystal nucleus.

Distinction between ring, shifted, and spindle particles

The observation that a significant proportion of SD clusters has an intermediate overlap with FCC or HCP during nucleation, raises the question which part of the SD cluster overlaps with FCC or HCP.

We divide the particles composing an SD cluster into three categories – *ring*, *spindle*, and *shifted* particles. The ring particles are the particles colored in light gray in Fig. 2b, and they are four of the five particles that make up the pentagonal ring of the PB cluster. The spindle particles are the pink particles that are bonded to all ring particles. These particles are also the spindle particles of the PB cluster. Finally, the shifted particles are the two pink particles on the right side of the SD cluster in Fig. 2b, and they replace one of the ring particles of the PB cluster.

In Fig. S3d, we plot the probability that a particle is part of an FCC or HCP cluster, given that it is part of an SD cluster with intermediate overlap with FCC or HCP. We plot these probabilities separately for ring, spindle, and shifted particles. The ring particles have a smaller probability of being in an FCC or HCP cluster than the spindle and shifted particles. This suggests that the SD clusters have a preference to be oriented such that the shifted particles point towards the center of the crystal nucleus. The shifted particles are the key difference between PB and SD clusters. Recalling that the disappearance of PB clusters and the subsequent excess of SD clusters enables the onset of the crystallization phenomenon, it is to be expected that the *shifted* particles of the SD cluster are indeed the first to crystallize.

Behavior of other clusters in nucleation, experiments, and coexistence

In the main text, we show that the OH, PB, and SD clusters are the most important clusters to consider for crystallization and polymorph selection, because they have the highest mutual information on these processes. However, for completeness, we also show the behavior of the other clusters. We excluded the 10W, 11A, 11B, 11W, 12K, 13A, 13K, and 10K clusters because they were extremely rare.

Behavior of other clusters during nucleation

In Fig. S4, we show the fraction of particles that are part of each cluster during nucleation. Most clusters show monotonic behavior, i.e. they either decrease or increase during nucleation. In this sense, the SD cluster is unique: it is the only cluster to show non-monotonic behavior during nucleation. Furthermore, Fig. S4 shows that the 'disordered' fluid phase actually contains many different locally favored structures due to packing contraints.

Comparison with experiments

We now make a quantitative comparison of the behavior of clusters between experiments and simulations. To this end, we first compute the number of solid-like bonds for each particle (see Methods). Then, for each number of solid-like bonds, we compute the average number of clusters a particle is part of. In Fig. S5, we plot the average number of clusters a particle is part of as a function of the number of solid-like bonds. We shade the region between 5.5 and 8.5 solid-like bonds in gray because these particles are most likely to be near the solid-fluid interface.[6] In the case the number of solid-like bonds for a particle is smaller than 5.5, the particle most likely belongs to the fluid phase, while a particle with 8.5 or more solid-like bonds belongs most probably to the solid phase. Similar to Fig. S4, most clusters show a

monotonic behavior, i.e. they either decrease or increase with the number of solid-like bonds, whereas the SD cluster is the only cluster to show non-monotonic behavior.

Overall, we see excellent agreement between experiments and simulations, especially for particles with fewer than eight solid-like bonds. With an increasing number of solid-like bonds, the agreement between experiments and simulations becomes somewhat worse. The main reason for this seems to be the slightly lower number of HCP clusters in experiments. The simulation data was taken from the nucleation trajectories, while the experimental data contain fluid, nucleation, and crystal growth data. For larger crystals, it is expected that fcc is more predominant than hcp because fcc is slightly more stable than hcp. Therefore, it is expected that the larger crystals in the experiments contain fewer HCP clusters than the data from simulations. The other clusters where differences are visible, e.g. 9K and 11F clusters, are clusters that are indeed associated with hcp crystals.[7]

Behavior of other clusters near planar solid-fluid interfaces

In Fig. S6, we show the behavior of clusters near an fcc-fluid or hcp-fluid interface. To this end, we simulate an fcc crystal with the 110 plane and an hcp crystal with the $11\bar{2}0$ plane in coexistence with a fluid phase. We use these planes because of their relatively low interfacial tensions,[8] and because there is no risk of stacking faults. In the case of the 110 (fcc) or 0001 (hcp) plane, stacking faults could occur when hcp grows on fcc, and fcc grows on hcp. We simulate $N \approx 7000$ particles interacting with a WCA potential (see Methods) at an effective packing fraction $\eta_{\text{eff}} = \pi \sigma_{\text{eff}}^3 N/6V = 0.517$.

As before, we see that most clusters show monotonic behavior. The SD cluster shows the strongest non-monotonic 'bump' around the solid-fluid interface. Additionally, the number of SD clusters is higher at the fcc-fluid interface than at the hcp-fluid interface, and also higher in the fcc crystal than in the hcp crystal. This reflects the preference of SD clusters for fcc. Apart from SD, there are some other clusters – 9K, 11F, 12E, and BCC9 – that show a slight non-monotonic 'bump' around the solid-fluid interface. All of these clusters also

have relatively high mutual information (MI) on crystallization and polymorph selection, as shown in Fig. 2. However, there are also clusters with high MI, such as OH, which just show monotonic behavior around the solid-fluid interface.

By comparing bulk fcc and hcp, we see that hcp contains significantly more sp3a, sp3c, 9K, 11F, 12E and BCC9, and fcc contains significantly more SD and BCC9. This was already observed by Malins et al.[7] However, the clusters that show the largest differences in bulk do not necessarily have the highest MI on polymorph selection. Comparing with Fig. 2, we see that sp3a and sp3c have a low MI. This may be caused by the fact that sp3a and sp3c are too common in the fluid, and thus the fluid has a homogeneous structure with respect to sp3a and sp3c. On the other hand, PB and OH have high MI, despite the fact that fcc and hcp contain roughly equal numbers of each. As discussed in the main text, this is because PB is closely linked to the SD cluster and its 'squared' variant, which does show a 2-to-1 preference of fcc over hcp. The OH cluster has a high MI because it competes with fivefold symmetry in the fluid. Whereas the fivefold symmetry favors fcc, the OH cluster has no preference. The drift to 50% results in a high MI for OH.

References

(1) van Meel, J. A.; Filion, L.; Valeriani, C.; Frenkel, D. A Parameter-Free, Solid-Angle Based, Nearest-Neighbor Algorithm. *Journal of Chemical Physics* **2012**, *136*, 234107.

(2) Lechner, W.; Dellago, C. Accurate determination of crystal structures based on averaged local bond order parameters. *Journal of Chemical Physics* **2008**, *129*, 114707.

(3) Stukowski, A. Structure identification methods for atomistic simulations of crystalline materials. *Modelling and Simulation in Materials Science and Engineering* **2012**, *20*, 045021.

(4) Larsen, P. M.; Schmidt, S.; Schiøtz, J. Robust structural identification via polyhedral

template matching. *Modelling and Simulation in Materials Science and Engineering* **2016**, *24*, 055007.

(5) Fiorucci, G.; Coli, G. M.; Padding, J. T.; Dijkstra, M. The effect of hydrodynamics on the crystal nucleation of nearly hard spheres. *Journal of Chemical Physics* **2020**, *152*, 064903.

(6) Filion, L.; Hermes, M.; Ni, R.; Dijkstra, M. Crystal nucleation of hard spheres using molecular dynamics, umbrella sampling, and forward flux sampling: A comparison of simulation techniques. *Journal of Chemical Physics* **2010**, *133*, 244115.

(7) Malins, A.; Williams, S. R.; Eggers, J.; Royall, C. P. Identification of Structure in Condensed Matter with the Topological Cluster Classification. *Journal of Chemical Physics* **2013**, *139*, 234506.

(8) Sanchez-Burgos, I.; Sanz, E.; Vega, C.; Espinosa, J. R. Fcc vs. hcp competition in colloidal hard-sphere nucleation: on their relative stability, interfacial free energy and nucleation rate. *Physical Chemistry Chemical Physics* **2021**, *23*, 19611–19626.

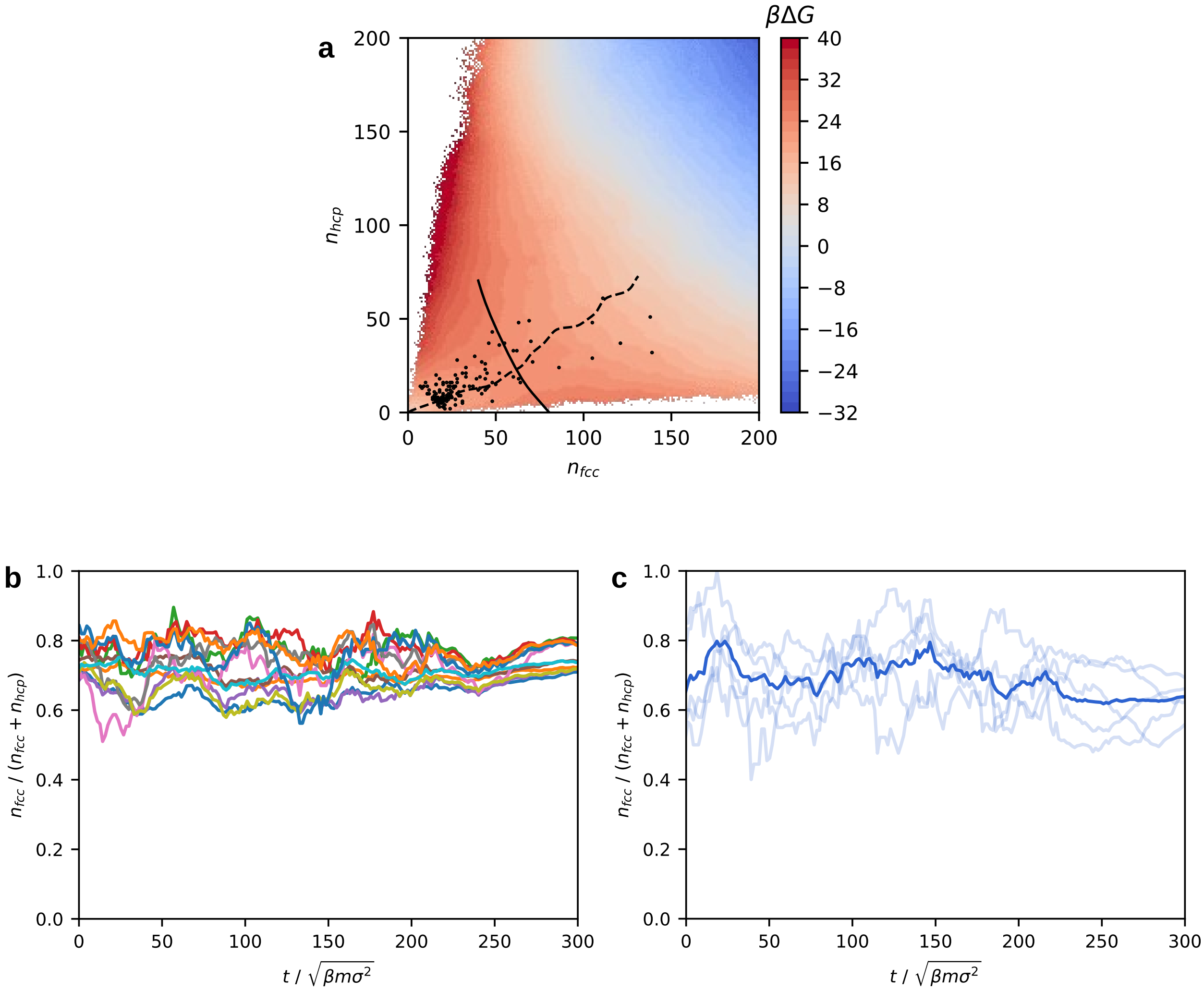

Figure **S1**: **Crystal polymorphs in the growing crystalline cluster.** (a) Gibbs free-energy barrier as a function of the number of fcc particles n_{fcc} and the number of hcp particles n_{hcp} as recognized by the classification scheme described in the *Methods* Section. The dots are the composition of crystal nuclei found in our experiments. The dashed line represents the minimum free-energy path for nucleation, and the solid line the critical nucleus size as a function of composition. (b) Relative fraction of fcc-like particles in the growing nucleus as identified by 12 different classification schemes described in the text. (c) Relative fraction of FCC clusters with respect to HCP clusters as identified by the topological cluster classification.

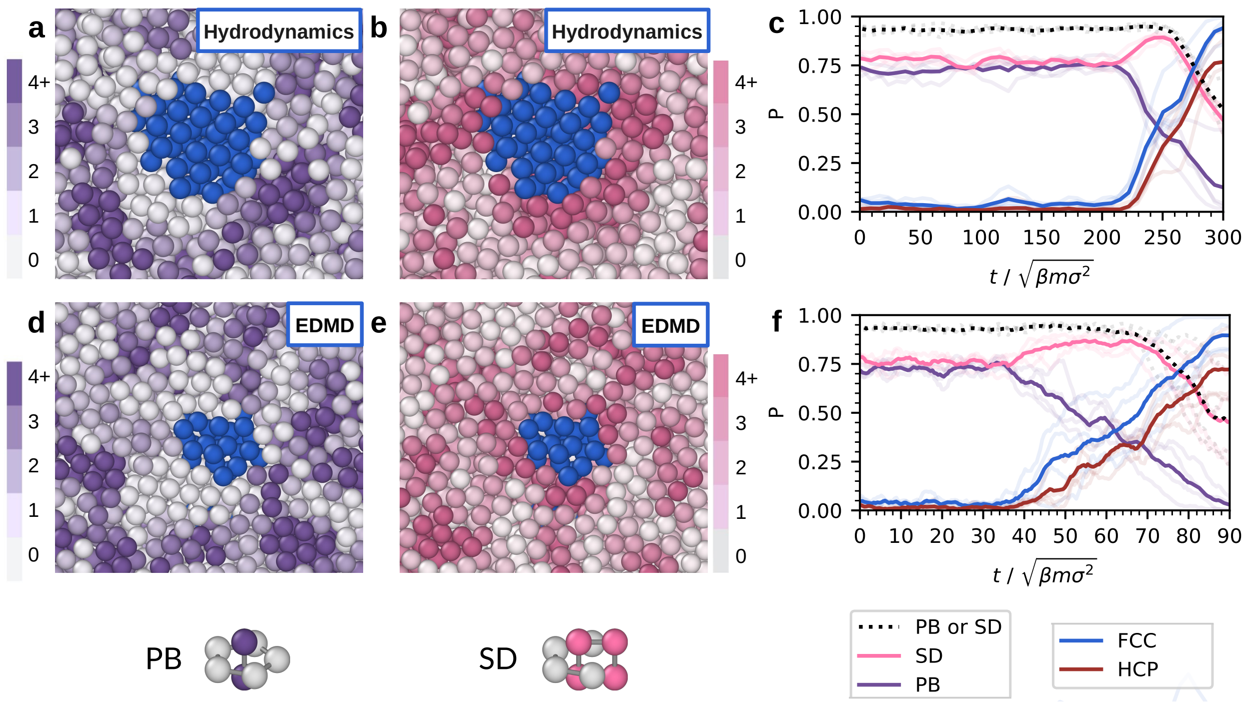

Figure **S2**: **Behavior of SD and PB clusters during crystal nucleation from strongly correlated, dense fluids.** (a-c) Nearly hard spheres modeled with a WCA potential using the Stochastic Rotation Dynamics method for hydrodynamics. (d-f) Hard spheres using event-driven molecular dynamics simulations. Please see the caption of Fig. 3 of the main text for further details.

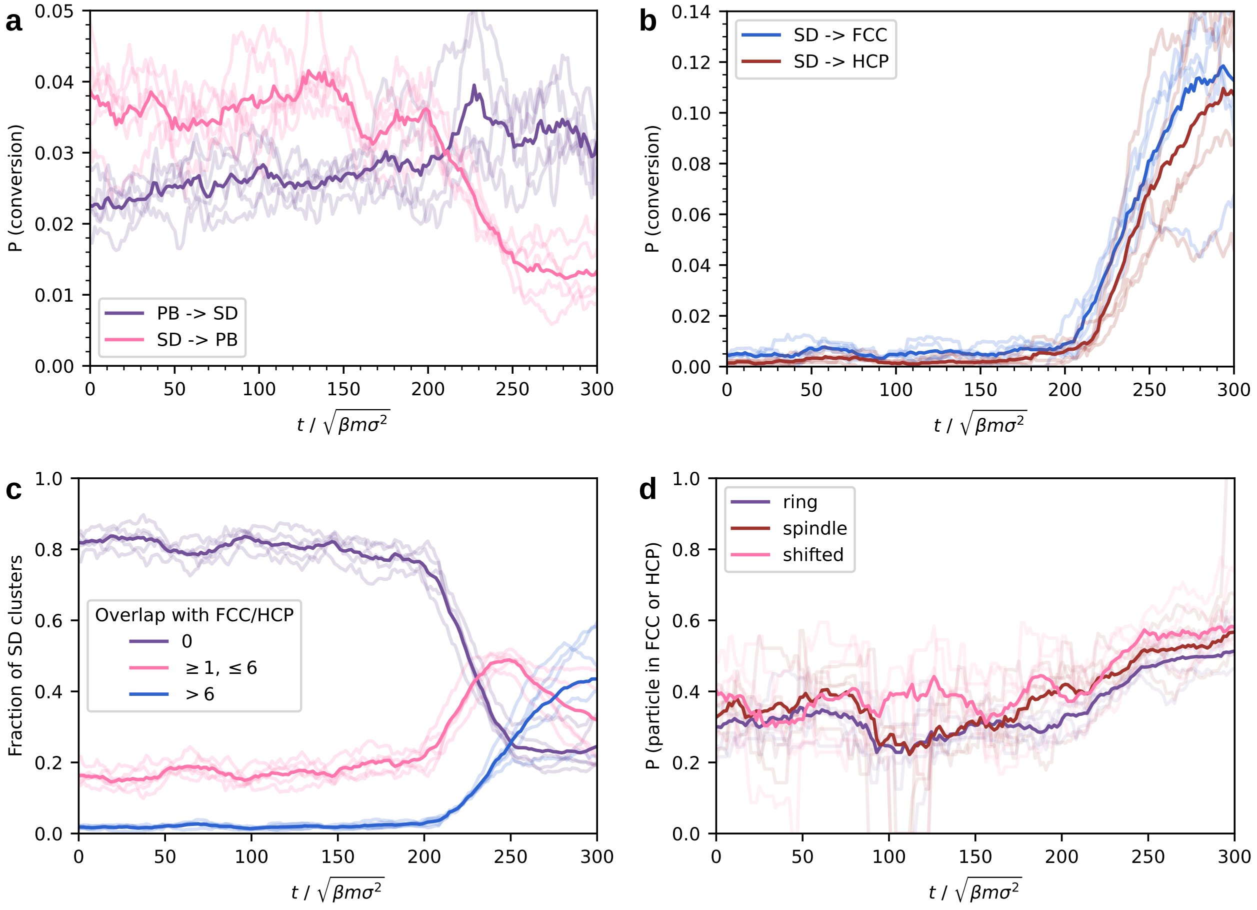

Figure **S3**: **Role of the SD cluster as an intermediate in crystal nucleation.** (a) Probability of conversion from PB to SD and from SD to PB clusters during nucleation. Please see the text for a definition of a conversion. (b) Probability of conversion from SD to FCC and from SD to HCP clusters during nucleation. (c) Overlap of SD clusters with FCC or HCP clusters during nucleation. (d) Probability for different constituent particles of SD clusters to also be part of FCC or HCP clusters during nucleation. Please see the text for a definition of ring, spindle, and shifted particles. Each plot contains multiple nucleation pathways, plotted with a transparency of 0.9. Each path is shifted in time such that the number of solid-like particles reaches 50 at approximately the same time. Solid lines are averages of these pathways.

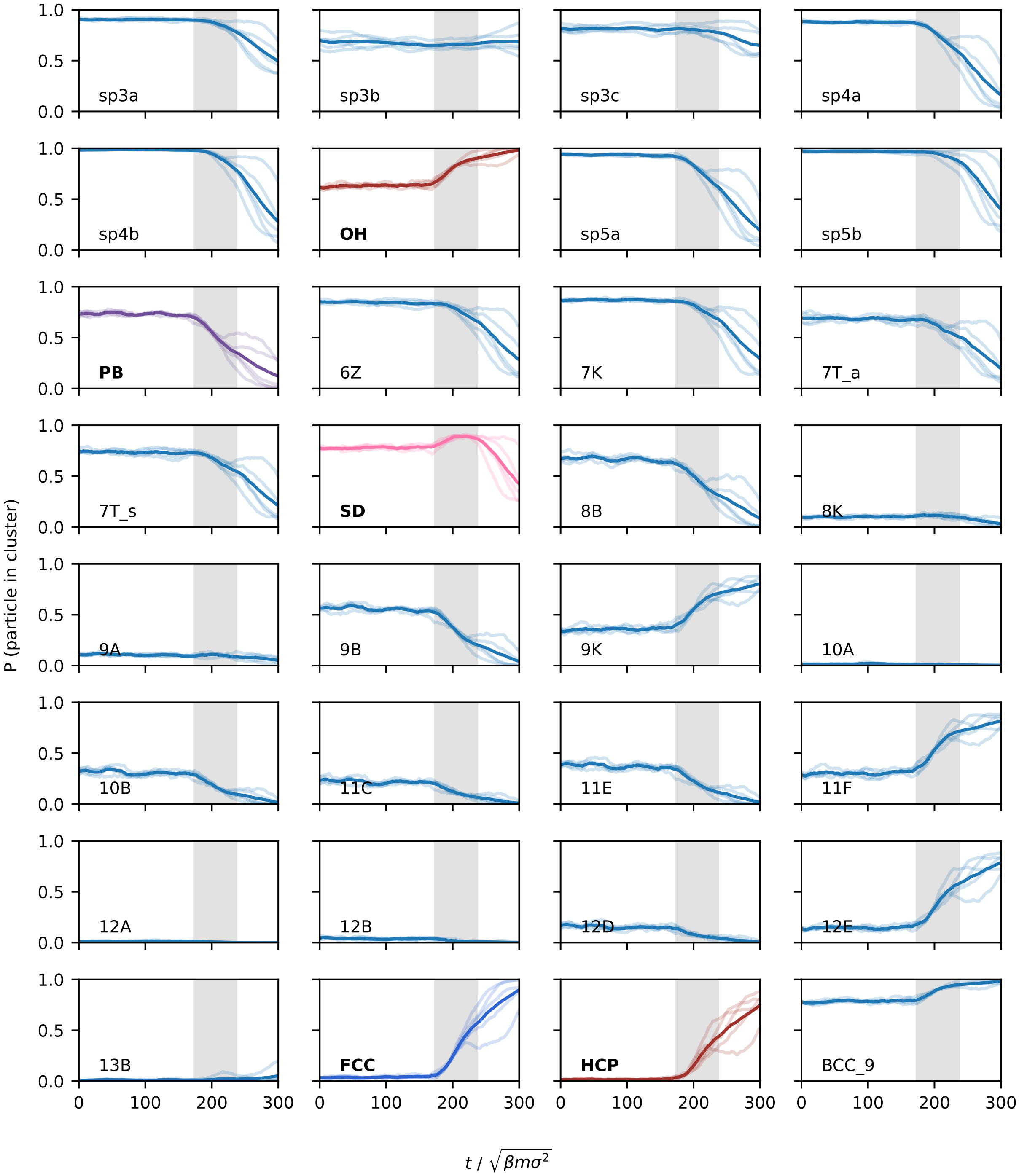

Figure **S4**: **Behavior of other clusters during crystal nucleation.** For each cluster identified by the topological cluster classification (except for 10W, 11A, 11B, 11W, 12K, 13A, 13K, and 10K), we plot the fraction of particles that is part of that cluster. Each plot contains multiple nucleation pathways, and the solid lines are averages over these pathways. We shade the area between $t/\sqrt{\beta m \sigma^2} = 174$ and $t/\sqrt{\beta m \sigma^2} = 237$ in grey to highlight the start of nucleation.

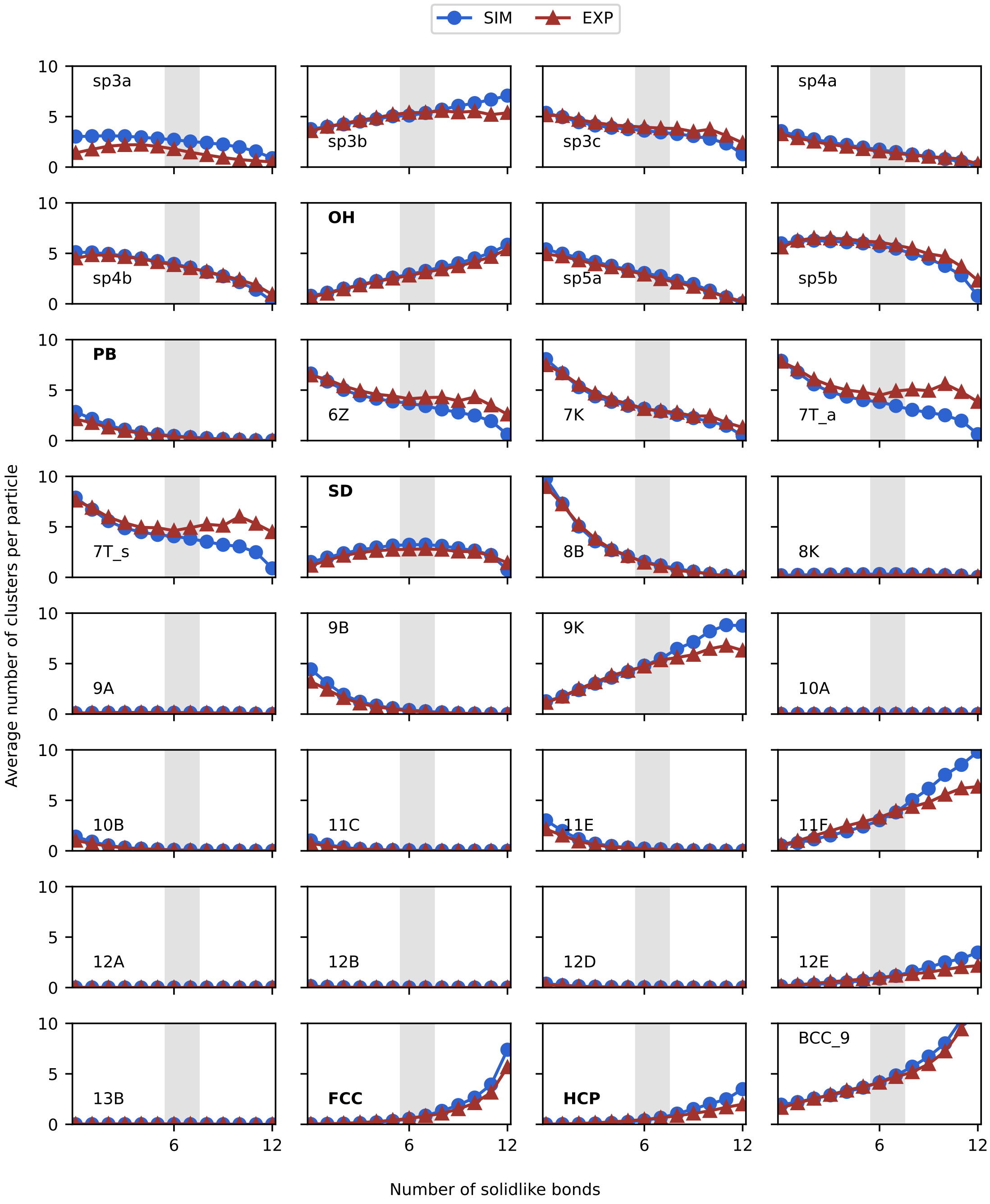

Figure **S5**: **Comparison between experiments and simulations.** For each cluster identified by the topological cluster classification (except 10W, 11A, 11B, 11W, 12K, 13A, 13K, and 10K), we plot the average number of clusters a particle is part of, as a function of the number of solid-like bonds of that particle. We shade the area between 5.5 and 8.5 solid-like bonds in grey to highlight particles that are most likely near a solid-fluid interface. To the right of the shaded region we have most likely the solid-like particles, whereas to the left we have most likely the fluid-like particles.

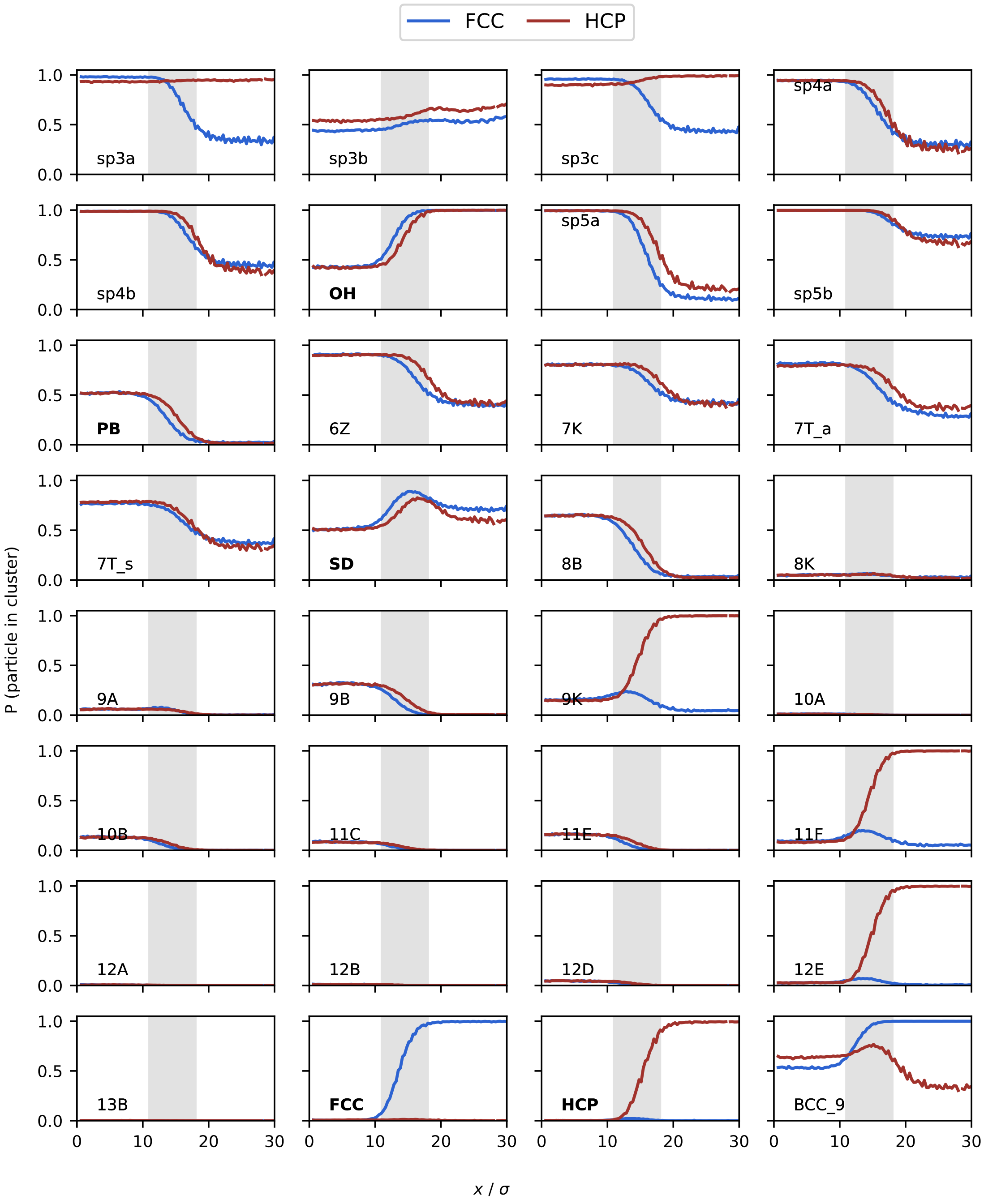

Figure **S6**: **Behavior of topological clusters near a solid-fluid interface.** For each cluster identified by the topological cluster classification (except 10W, 11A, 11B, 11W, 12K, 13A, 13K, and 10K), we plot the fraction of particles that is part of that cluster, as a function of the coordinate perpendicular to a solid-fluid interface. In blue, we show fcc in coexistence with the fluid, while in brown, we show hcp in coexistence with the fluid. We shade the area between $x/\sigma = 11$ and $x/\sigma = 18$ in grey to highlight the solid-fluid interface. To the right of the shaded region we have the solid, whereas to the left we have the fluid.
